# Perceived impacts as narrated by service users and providers on practice, policy and mental health system following the implementation of the mhGAP-IG using the TEAM model in a rural setting in Makueni County, Kenya: a qualitative approach

**DOI:** 10.1186/s13033-019-0309-4

**Published:** 2019-08-14

**Authors:** Victoria N. Mutiso, Christine W. Musyimi, Tahilia J. Rebello, Michael O. Ogutu, Ruth Ruhara, Darius Nyamai, Kathleen M. Pike, David M. Ndetei

**Affiliations:** 1 Africa Mental Health Research and Training Foundation, Matumbato Road, Mawensi Gardens, Upper Hill, Nairobi, Kenya; 2 Global Mental Health Program, Columbia University, New York, USA; 3 Department of Psychiatry, University of Nairobi, Nairobi, Kenya

**Keywords:** mhGAP-IG, Mental health system, TEAM, Perceptions, Narratives, Practice, Policy, Kenya

## Abstract

**Background:**

A team approach is key to reduction of the mental health treatment gap. It requires collaborative effort of all formal and informal community based service providers and the consumers of the services. Qualitative evaluations of such an approach add value to the quantitative evaluations.

**Methods:**

A qualitative study was conducted among 205 participants. These were grouped into a total of 19 focus group discussions for community health workers (CHW), traditional healers (TH), faith healers (FH) and patients. For nurses and clinical officers we held 10 key informant interviews and three key informant discussions. We aimed to document personal perceptions as expressed in narratives on mental health following a community based application of the WHO mental health treatment Gap-intervention guideline (mhGAP-IG) using the TEAM model. We also aimed to document how the narratives corroborated key findings on the quantitative wing of the TEAM model.

**Results:**

There were three categories of perceptions: (i) patient-related, (ii) health provider-related and, (iii) health system related. The patient related narratives were linked to improvement in their mental and physical health, increased mental health awareness, change in lifestyle and behavior, enhanced social functioning and an increase in family productivity. Health provider perceptions were related to job satisfaction, capacity building and increased interest in mental health training. Mental health system related narratives included effectiveness and efficiency in service delivery and increase in number of referrals at the primary health care facilities.

**Conclusion:**

The TEAM is a feasible model for the implementation of the mhGAP-IG. It led to positive perceptions and narratives by service provides and service consumers. The qualitative findings corroborated the quantitative findings of TEAM.

## Background

Health systems in low and middle income countries (LMICs) are yet to adequately respond to the burden of mental health problems [1, 2]. Up to 85% of those with severe mental disorders in LMICs receive no treatment for their disorders [2, 3]. LMICs also receive little financial allocation for mental [4, 5] because the national health priorities focus on communicable diseases, such as malaria, HIV and AIDS and tuberculosis [6]. Kenya has only 1–2 primary health care workers per 10,000–20,000 people, and no medical doctors at the primary public health care level except nurses and clinical officers [6]. Currently, the psychiatrist: population ratio is 1:5000,000, with the psychiatrists working in or near major cities [7]. Both traditional healers (TH) and faith healers (FH), commonly referred to as Traditional Health Practitioners (THPs), are often times the first line of contact for individuals suffering from mental disorders, not only in Kenya but other African countries [8, 9] as also acknowledged by WHO mhGAP-IG [10]. THPs form part of the community’s cultural belief system, are flexible in terms of modes of payment (not necessarily on monetary terms), are available within the communities and are accessible on demand [11]. Community Health Workers (CHWs) also link the community and health facilities but have no formal training on providing mental health services [12]. Together, the THPs and CHWs comprise the complementary health sector. THPs and CHWs have the potential to be a link in the integration of mental health services to primary health care level for better mental health outcomes [1, 13, 14]. Interventions in primary care settings allow for early detection and management, potentially enhancing clinical outcomes, and reduced treatment costs [15].

It has been reported that stigma and discrimination can be reduced when seeking mental health care from a primary health care provider compared to a mental health specialist [1, 16].

It was from the above perspective that we initiated a study aimed to reduce the mental health treatment gap in Kenya using the already available formal health care workers (nurses and clinical officers) and informal health providers (TH, FH, CHWs) using a task-sharing approach. To achieve this, we developed a model “Multisectoral Stakeholder TEAM Approach to Scale-Up Community Mental Health in Kenya—Building on Locally Generated Evidence and Lessons Learned (TEAM)”. TEAM sought to identify and bring together through participatory dialogue the already existing community based human resources which could be trained to provide mental health services in the community. It also intended to do this using already available systems, financial and infrastructural resources. This TEAM model has been detailed in one of our papers [17]. In summary, we conducted an analysis of mental health services in Makueni County, one of the 47 counties in Kenya, in order to understand the existing gaps and opportunities in a low-resource setting. We undertook a stakeholder analysis and engagement through interactive dialogue in order for the stakeholders to appreciate the importance of mental health to their communities. Through the process of participatory Theory of Change, the stakeholders gave their inputs on the process between the initiation and the end of the process for community mental health development. We aimed to achieve buy-in and collective ownership of the whole process. We adapted the mhGAP-IG [10] to the local context. We trained local human resources in skills necessary for the implementation of mhGAP-IG and for monitoring and evaluating the process using instruments with good psychometric properties that have been used in LMICs. Faith healers (FHs), traditional healers (THs) and CHWs were trained, each cadre separately, to screen for common mental disorders using the mhGAP-IG. They were also trained to refer to nurses and clinical officers who had been trained to provide management including biological management (psychotropic drugs) using the mhGAP-IG. Nurses and clinical officers who had not been trained did not receive referrals for the purpose of TEAM. Using this multi-stakeholder approach which included policy makers, we demonstrated the feasibility of implementing the mhGAP-IG using existing and trained community human resources and also the feasibility to transit seamlessly from research to policy and practice uptake [17]. In a different publication, we reported that both nurses and clinical officers as well as CHWs, THs and FHs can be trained to successfully and accurately screen for mental health disorders using the mhGAP-IG symptoms and that they may play a key role in decreasing the mental health treatment gap [18]. There were high concordances in the accuracy of screening by all the service providers for both formal and informal [19]. In yet another publication we reported significant reduction of experienced discrimination in people with mental illness on the use of the mhGAP-IG face-to-face psychoeducation with the aim of reducing stigma [20]. We have also demonstrated significant increase in mental health related knowledge in people with mental illness using the mhGAP-IG [21]. More recently we demonstrated that a combination of psychoeducation of people with common mental disorders and of nurses and clinical officers who are trained on mhGAP-IG, supported and supervised can lead to improved outcomes in disabilities, quality of life and clinical outcomes in people with common mental disorders [18]. Overall, our above publications on the quantitative phase of TEAM have demonstrated significant and positive outcomes on the mhGAP-IG based interventions.

The aim of this qualitative phase was to document what the patients and service providers perceived to be the impacts of the implementation of TEAM. We also aimed to find out if those perceptions on TEAM corroborated the quantitative outcomes of TEAM. We sought to do this by teasing out their perceptions, expressed in their own words in the form of verbatim narratives in the following areas: (1) patients’ own perception on physical and mental health, awareness in mental health, life style and behavior, social functioning, family productivity, earning and savings; (2) health provider perceptions on capacity building, job satisfaction, mental health training; (3) health systems-related perceptions by either patients or service providers on effectiveness and efficiency in service delivery, referrals.

## Methods

This was a qualitative cross-sectional study conducted among 205 participants.

A total of 19 focus group discussions (FGDs) were conducted, each with 8–12 participants comprised of Informal Health Providers (IHPs) (four with CHWs, three with THs and four with FHs). These FGDs for different IHPs were conducted separately and independently so as not to allow any influence by the other group of their perceptions. We also conducted eight FGDs for service users [18]. We based our number of FGDs on recent evidence that showed that between three and six FGDs are adequate to identify the most prevalent themes within datasets [22]. Four key informant discussions (KIDs) (participants ranging between 3 and 6 participants) and 10 key informant interviews (KIIs) were conducted with clinicians. Since nurses and clinical officers are relatively unavailable at the same time for purposes of FGDs, we conducted only KIDs and KIIs for them.

The recruitment for the FGD participants was from the service users. These included patients with common mental disorders listed in the mhGAP-IG but confirmed for DSM-IV/ICD 10 diagnosis using the MINI-Plus [18]. These are the patients who had taken part in the quantitative phase of the study. We also included service providers who had taken part in the implementation of the study. We included participants who were available on the day of the interviews and gave informed consent.

All the interviews were conducted in the privacy of rooms at the health care facilities. The patients had been mobilized by the CHWs to come to the facility. As for the FHs and THs, we hired rooms in the locally available community facilities where we had trained them at the start of TEAM. The interviews were conducted by facilitators with a minimum qualification of an undergraduate degree in a health-related field and at least 2 years of experience in community mental health research. They had undergone 3 days of training by one of the authors (MOO) who has a Master level degree in qualitative research training. They were trained on how to engage and conduct interviews using the COREQ guidelines [23].

Due to lower literacy levels amongst the IHPs, their interviews were conducted in the local languages “Kiswahili” or “Kikamba”. For clinicians and CHW’s interviews were conducted in English. Transcriptions and translations were conducted by a linguist fluent in the local languages and checked for consistency by an independent linguist. The discrepancies were resolved through a discussion among a team of linguists, health and non-health teams before a final version was agreed upon. We endeavored to reproduce verbatim what was said by different respondents. We only did minimal editing on punctuation and clarification (in brackets) for clarity of flow. Written informed consent was obtained from all participants before data collection and ethical approval was granted by the Maseno University Ethics and Review Committee.

### Data analysis

All transcribed and translated data was managed using NVivo qualitative software version 10. We used content analysis by initially coding the data line-by-line and thereafter constructed a thematic framework to link codes to certain themes before final interpretation.

After reviewing the field notes and transcripts, the research team met for a day where they used an iterative approach in order to agree upon which core ideas and concepts were repeated across the interviews as well as what themes emerged more peripherally. Several themes and subthemes were identified. Once the list was agreed upon, the 2nd, 3rd and 5th authors developed a codebook with explicit definitions of each theme. The third and fifth authors carefully coded narrative interviews. The rest of the authors reviewed codes to ensure that the codes and text corresponded and reflected the definitions generated by the research team. Exemplar quotations were excerpted to illustrate what themes emerged from the narratives.

## Results

Table 1 summarizes the different types of study participants, types of interviews and numbers interviewed. All groups of service providers in the community were represented. The patients–the recipients of the services were also represented.

**Table 1:** Data collection methods used in the study

Method of data collection	Target group	Number of interviews	Number of participants per interview	Total number of participants
Focus group discussion	Community health workers (CHWs)	4	8–12	43
	Traditional healers (THs)	3	8–12	25
	Faith healers (FHs)	4	8–12	32
	Patients	8	8–12	77
Key informant interviews	Clinicians	10	1	10
Key informant discussions		4	3–6	18

**Fig. 1:**
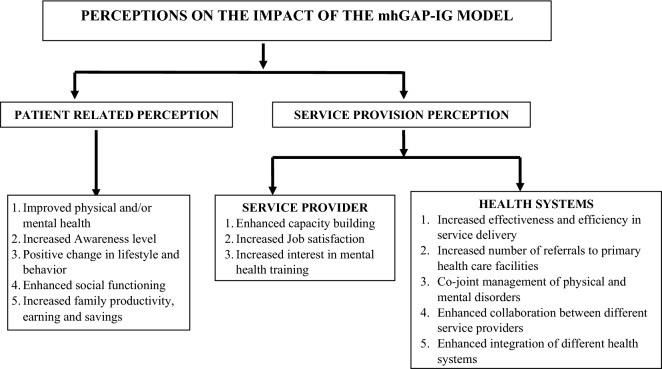
Perceptions associated with integration of health services in primary care settings

Figure 1 summarizes the themes of what the different interviewees perceived to be the impact of the implementation of TEAM. These can be summarized under several themes.

### Patient-related perceptions

#### (i) Improved physical and/or mental health

A number of patients suffering from different mental disorders verbalized improvements in dealing with their daily struggles as well as improvements in physical conditions that co-existed with the mental disorders. For instance, those suffering from depression were able to manage their stress levels after receiving psychosocial interventions at the local health facilities, which resulted in reduction in their blood pressure levels.

**Narrative 1:**

*“I have seen change because since I started receiving counseling from the doctor [clinician], there is change in my body. I don’t often suffer from pressure (high blood pressure) like I used to. The doctor even reduced my treatment dosage. So that is a big signal (positive response) resulting from the counseling I received”* *(34* - *year* - *old male patient).*

**Narrative 2:**

*“There is another one (patient) who was depressed and he even got high blood pressure because of the depression but right now he is fine and his pressure is down…he tells me that these days he sleeps and eats well and he’s able to manage his stress”* *(30* - *year* - *old female CHW).*

#### (ii) Increase in awareness level

Before the TEAM approach was rolled out, the community viewed mental health problems as a curse or a result of witchcraft [24]. They thought that mental health problems were incurable. Thus they were hesitant to visit clinics. Community service providers tried their usual modes of treatment, which were not consistently successful. This often resulted in frustration and poor patient outcomes due to a lack of adherence to treatment.

**Narrative 3:**

*“Before we got this training, most of the community members saw the different mental problems as a curse. They therefore saw no need of going to the clinic (for treatment). Before we [faith healers] were trained, they [the patients] just met us in church and we could just pray for them, sometimes the prayers worked and sometimes the work was not easy. The people thought that those with mental problems got them because of witchcraft which could be passed down ones’ linage (were inherited)”* *(58* - *year* - *old male faith healer).*

However, with the TEAM approach in place, respondents noted an increase in community awareness levels which they associated with the training provided to IHPs. The increase in awareness levels was also attributed to local mass media campaigns, and local public sensitization meetings [17, 25]. Many community members gained cognizance of mental health treatment, as cited below by a male faith healer in his late sixties.

**Narrative 4:**

*“This intervention helped people to know that some diseases that people felt could not be cured were indeed curable. This was noted when those who were known to be mentally ill [patients], came back from hospital and told others that they were treated and that many who went to the hospital had improved. For instance, there was a man who was a known drunkard. I referred him to the clinic and was treated for six months. These days, he is sober and people came to realize that alcohol abuse can be cured”* *(A faith healer in his late sixties).*

Awareness levels also increased because of home visits made by IHPs as illustrated by a CHWs speaking on behalf of informal service providers.

**Narrative 5:**

*“Initially before this intervention (TEAM) people did not know where they could get help. Now that we [CHWs, and THs and FHs] have now visited them and talked to them, they know where they can get help when they fall ill”* *(36* - *year* - *old female CHW).*

The community got to know that mental illness was a disease like any other and not necessarily attributed to a curse or witchcraft. This resulted in some patients seeking treatment for mental illness for the first time.

**Narrative 6:**

*“Those who used to go for treatment from the traditional healers have also changed their attitude towards people with mental illness. They now know that when someone is mentally ill, they can receive treatment from the health facility”* *(39* - *year* - *old female faith healer).*

#### (iii) Change in life style and behavior

Both formal (clinicians) and informal health providers (THs, FHs and CHWs) noted a change in lifestyle and behavior among the community members (service consumers) with most of them opting to reduce or stop drug and substance abuse.

**Narrative 7:**

*“This intervention (TEAM approach) has really helped people in my community. For instance, there was a person who used to drink a lot of alcohol to try and forget his problems. He would even be washed by people in public because he was unkempt. He did not have a wife, but now I can say he is doing well* – *he has a wife. He doesn’t drink like he used to and he is clean…”* *(49*-*year*-*old female CHW).*

**Narrative 8:**

*“The number of people who were chewing miraa [khat] (a locally produced psycho*-*stimulant plant) and also abusing other drugs has reduced. If you talk to the patients, some have stopped taking alcohol and are doing well”.* *(28*-*year*-*old female clinician).*

#### (iv) Enhanced social functioning

Treatment of mental disorders at the primary health care level boosted the ability of the patients to play meaningful roles in the family by enhancing their social functioning. Patients reported feeling relieved and more confident to interact with other people and take part in community activities, as illustrated by one of the male patients.

**Narrative 9:**

*“… I never used to be comfortable near people because when I fall [get a seizure], someone may take me wrongly. But I now feel free to mingle with other people. You know I sing at the (church) choir spreading the gospel (Christianity).I now repair shoes. Although there was a day I felt dizzy and sat by the road because I did not take my medicine for a day, I now know how to take my medicine and I have really benefited from this.* (*A male epileptic patient).*

#### (v) Increased family productivity, earnings and savings

An increase in productivity and earnings at the family level was reported by the patients.

**Narrative 10:**

*“some families have reported increased earnings since those family members who were previously sick can now work”* (A male traditional healer).

**Narrative 11:**

*“I have had a change because I never used to take a bath. I was also not able to lift the hammer to repair containers or tanks unless I had taken at least half or a quarter glass of alcohol. But now I can take a month without taking alcohol. The customers also now appreciate me because before I was not able to attend to them and would therefore not make any earnings. But nowadays, I can work and at the end of the day, go back home with something for my family”* (48-year-old male patient who practiced as a self-employed artisan)

Other patients also reported an increase in savings due to reduction in costs spent in receiving treatment from distant health facilities (They could get help at the community health facilities since we had trained the nurses and the clinical officers. Through involvement of the Ministry of Health officials, psychotropic drugs were made available at these facilities).

**Narrative 12:**

*“Yes, there are also changes in the family income, since we [those with mental health conditions] no longer spend a lot (money) going for treatment. The services and drugs are now readily available at the health center”* (A female patient).

### Health provider related perceptions and narratives

#### (i) Capacity building

Both formal and informal health providers felt empowered after receiving training on mhGAP-IG. This allowed them to make better informed clinical decisions and provide appropriate mental health treatment themselves instead of referring patients to distant hospitals;

**Narrative 13:**

“*…at times, you come across these patients with perhaps psychosis or epilepsy at the health center, but you could do very little to assist. Therefore we always referred them to the County referral hospital for mental health services but right now I am comfortable to assist them here (at the health centre)”* *(28*-*year*-*old male clinician).*

A 47-year-old female traditional healer also described how the training increased her knowledge and skills on detecting mental illness e.g. comprehensive history taking, and referral strategies. This helped the TH to enlarge her clientele and in the process gained financially:

**Narrative 14:**

“*This training enlightened me and I am now better placed to tackle the different mental conditions besides psychosis which was easy to tell. I also get more clients than before.”* *(A 47*-*year old female traditional healer)*

#### (ii) Job satisfaction

The clinicians also reported a sense of emotional reward on seeing the impact of their work. Patients reported improvements during return visits, motivating clinicians to continue providing treatment to patients suffering from similar conditions.

**Narrative 15:**

*“…I had a patient who had a history of seizures and classical signs of epilepsy, if I may say so. I prescribed drugs for one week as a start and advised them to go try it out. Fortunately, the girl only had one seizure after the start of the treatment. They even came back before the return date since they saw that things were working. I therefore gave them the rest of the remaining dosage. This change really encouraged me and I think she is one of my favorite patients because when I see her, I see the positive outcome and feel like helping out more”* *(28* - *year* - *old male clinician).*

#### (iii) Increased interest in mental health training

The demand for mental health services at the primary health care level was manifested by the increased number of referred cases. This made the Community health workers feel a need to go for mental health training whenever the opportunity arose. There was a shortage in health care providers at the primary health care level. This was a call for policy change in the training of more mental health workers coming from health workers themselves.

**Narrative 16:**

*“We know that very soon because of the increase in numbers, the facilities will need people who are trained on mental health to assist you people. So, we will be grateful if you also consider us (for further training)…”* *(32* - *year* - *old Female CHW)*

### Health system related perception and narratives

#### (i) Increased effectiveness and efficiency in service delivery

The TEAM approach increased the quality of service delivery. The services were more focused on particular mental health problems rather than providing general health care. It also enhanced efficiency in the use of the available resources. The health providers could appropriately prescribe drugs that were previously (in stock) at the primary health care level but not in use before.

**Narrative 17:**

*We [clinicians] have learned how to manage those patients at the health center level. Previously we would see those drugs but we did not understand which people should be given those drugs. Now we can give them comfortably”* *(30* - *year* - *old female clinician).*

The referral system was also strengthened through use of IHPs. Their role in the system was established, and they reported increased comfort in conducting follow-up appointments to check the progress their patients were making.

**Narrative 18:**

*“…we are now better placed to identify their [patients’] problems and refer them to hospital and even make follow*-*ups to see if they got better”.* *A 49*-*year*-*old female traditional healer*

The approach resulted in different stakeholders working collaboratively, streamlining the referral process and enhancing the effectiveness of service delivery.

**Narrative 19:**

*“As the research is called “Team,” it brought team work, because one thing we discovered with mental illness is that it is not a one*-*man thing. You cannot manage it alone. You can be good at diagnosing but you are not good at counseling the patient so you would diagnose and send the patient to someone who can counsel the patient. And also in prescribing and dispensing drugs, it is something that we had to do together. So, it brought about team work”.* *(30*-*year*-*old female clinician).*

#### (ii) Increase in number of referrals

The integration of mental health services via a clear referral and linkage system resulted in a reported rise in the number of referrals to local facilities for patients suffering from mental disorders.

**Narrative 20:**

*“…as a result of the program, I now get more clients than before whom I now refer to the hospital to get help”.* *(76* - *year* - *old female traditional healer).*

The increase in numbers of patients being seen for mental disorders could be attributed to the sensitization and defaulter tracing during home visits conducted by the IHPs, as cited below, by two male clinicians respectively.

**Narrative 21:**

*”…The numbers are increasing because the information is going deeper in the community”.* *(55* - *year* - *old male clinician).*

**Narrative 22:**

*“Before this intervention (TEAM) was in place, the community neglected their children and there was no tracing. Therefore because of the CHWs, THs and FHs, they were able to trace them and advise their relatives to bring them to the hospital (health facility)”* *(30* - *year* - *old male clinician).*

Participants also attributed the rise in referral numbers to the snow-balling effect of patients who improved after seeking treatment from health facilities.

**Narrative 23:**

*“… CHWs referred patients to us and we gave them [patients] the appropriate treatment. These patients would then go and come back with other patients with similar conditions so you find that there was some acceptance from the community that things were working out”* *(28* - *year* - *old male clinician).*

## Discussion

We report a study that, as far as we have been able to establish through global literature, is the first qualitative study from a LMIC to report the following all happening concurrently in one study: (1) personal perceptions as captured by narratives of patients and different types of community based non-mental health specialties; (2) the stakeholders working collaboratively and as a team in a community based setting; (3) using the mhGAP-IG as a common tool for all of them; (4) using concerted effort to build collectively owned human resource for mental health and (5) maximizing existing community owned human and financial resources for mental health. This study is not about the outcomes or efficacy of the mhGAP-IG by different health providers—we have already reported these in several quantitative studies and summarized them in the introduction [18–20, 26]. Instead, this was on how the application of the mhGAP-IG, using the TEAM model, had personally touched the lives of the patients and on the experiences of the service providers, expressed through verbatim personal narratives. We attach significance to our findings in that personal narratives reflect lived life experiences which are more powerful in the minds of users and service providers than statements of quantitative outcomes without personal touch or relevance. Perceptions can make or break stigma, one of the most important barriers to mental health uptake and one of the main causes of mental health treatment gap as pointed out by WHO [27] and also in some of Kenyan studies [28, 29].

This qualitative study focused on how service users and service providers perceived TEAM and how these corroborated earlier published qualitative outcomes of TEAM. Although we found positive perceptions and corroborations of the findings on the quantitative phase of TEAM, all of these are subject to caveats that place the TEAM model in both theoretical and practical contexts. As pointed out by WHO [10, 30], different contexts vary in terms of culture, practice, policy, human and financial resources that have a bearing on an appropriate health system model. This means different contexts will need to develop their own models or improve on those that they already have. Alternatively they can adopt other models that have been successfully piloted in different but contextually similar settings. Results from different contexts may not be applicable in another given context. In the case for Kenya, we developed the TEAM as an inclusive model of as many stakeholders and players that are already in existence and available.

The TEAM pilot model takes cognizance of the reality of minimal public resources for mental illness [31]. Therefore TEAM sought to maximize the use of the already available financial and human resources in the development of a mental health system. To achieve this, we sought to dovetail in a health system that has both informal (THs, FHs, CHWs) and the formal (nurses and clinical officers) systems. But it is a system that still largely excludes mental health as an ongoing concern, despite the fact that FHs and THs are still extensively consulted out of choice by patients, including those with mental disorders [32].

The TEAM model also borrows from WHO which recognizes and recommends a multi-stakeholder approach to mental health system development that includes both formal and informal systems. This is aptly captured by the WHO Mental Health Action Plan (2013–2020) when talking about multi-stakeholder approach to mental health: “….Greater collaboration with “informal” mental health care providers, including families, as well as religious leaders, faith healers, traditional healers……, is also needed” [30]. If TEAM as pilot model is successful, it could be adopted and piloted in other contexts. With the above caveats, we can now discuss our findings.

That the patients presented with a combination of physical conditions and mental disorders as illustrated by depression and high blood pressure suggests the need to integrate physical and mental health management even at primary health care as was the case in this study. The application of counseling (in this case mhGAP-IG based), not only led to perceived clinical improvement on the physical aspect but also the reduction of the doses of the drugs used for the physical conditions (Narratives 1 and 2). We mention depression and high blood pressure just as an example of the well-known co-morbidity of mental disorders and physical conditions [33]. These qualitative narratives also lend support to our quantitative data on TEAM that a combination of psychoeducation and physical treatment was widely applied by the clinicians in the course of TEAM, leading to multiple positive outcomes on disabilities, quality of life and clinical outcomes in the patients with mental disorders [18].

The shift from the traditional perception of mental illnesses as caused by a curse or by a religious cause (Narrative 3) has implications on paradigm shift on perception to the effect that such patients could be managed as medical cases. This kind of cooperation is indeed anticipated by the mhGAP-IG which states: “…Ask the person to let you know if they are seeing a traditional or a faith healer, showing respect for this, but emphasizing the need for being seen at a healthcare facility….” [10].

This is a potentially significant development given that THs and FHs are a first contact in health seeking behavior in Kenya [11], other countries in Africa [8, 9] and indeed many other countries [10]. This is a major step from stigmatization to acceptance of mental illness. It confirms our earlier quantitative findings that our approach had led to significant positive changes in perceived stigmatization amongst patients with mental illness [26]. A combination of service providers with a less stigmatizing attitude to patients with mental disorders, and patients feeling less discriminated has the potential to reduce the treatment gap from its current high level of about 85% in LMICs [10]. That this is feasible has already been urged in yet another quantitative publication arising from TEAM in which we demonstrated that THs, FHs and CHWs had increased referrals of cases of mental illness to the health facilities [18]. A significant development was the breaking of barriers between TH and clinicians in that the TH were able to recognize mental illness and refer to health facilities (Narratives 4, 6, 18, 20). This confirms our earlier findings that THs, FHs, nurses and clinical offices were willing to collaborate and cooperate and also referred patients to health facilities [19].

The overall impact of this concerted effort by key players in service providers in the community to identify and refer patients towards services including services at health facilities has also the potential to reduce the treatment gap [19]. Our approach (TEAM) suggests enhanced community connection that involved patients, the families and the communities (Narratives 7–12). This enhanced relationship and economic participation (Narrative 10–12) particularly at family level, and community acceptance of people with mental illness happened because they could see they (the patients) improved on treatment. This, in the process, reduced stigmatization. These qualitative narratives support the commonly accepted association between mental illness and poverty [34].

All the service providers appreciated the capacity building, which involved training them on the application of the WHO-mhGAP-IG. All of this is captured in Narratives 13–14 and throughout all the narratives on health system related perceptions (Narratives 17–23). As a result of this enhanced capacity they had more job satisfaction (Narrative 15). They also developed motivation for further training in anticipation for more numbers coming forward for treatment (Narrative 16).

There is the possibility that increased turnover of patients seeking for help from service providers who were empowered with skills could lead to overburdening and fatigue in the service providers [19]. Instead and as was found in this study, it led to enhanced job satisfaction and the desire to provide more help (Narrative 15). There was also a positive expectation to see more (Narratives 16, 23). There was the potential to avoid the revolving door phenomena for patients who would keep on coming back for conditions that were not properly diagnosed and managed (Narrative 17). If anything it would lead to a less burden and also a reduction on unnecessary overuse of services.

The training enhanced self confidence in the primary healthcare providers (Narratives 15, 17). This means they were able to reduce the number of referrals to higher level hospitals for the attention of doctors (Narrative 12). The patient who associated referrals to a hospital rather than a community based facility with high costs can be easily understood in the local context. Hospitals are considerable distances from the homes. Going to hospitals takes time and financial resources from family. It takes just about a day to travel from home to hospital (including waiting time) either walking long distances and/or paying for the expensive public or even private transport. This would normally involve at least two people—the patient and another person to accompany the patient to the hospital—that is two people whose economic activities are interfered with. The service providers were positively encouraged by the improvement of their patients. Noteworthy is that this encouragement led the health providers to want to help more people (Narratives 16, 23) as opposed to complaining about over-burdening. It led to increased interest in mental health training in order to meet the increased demand for services (Narrative 16).

TEAM brought about improvement in the health system that now accommodated mental health, to identify, manage and refer them if need be. It brought out the spirit of team work, where several stakeholders complemented one another and therefore it benefited everybody. In particular, the traditional healers clientele grew (Narrative 14), their incomes increased while at the same time, they referred more patients to the healthcare facilities. Therefore cooperation between traditional healers and the formal sector is feasible as was demonstrated in an earlier paper [24]. It does not necessarily reduce the traditional healers’ incomes; rather it could potentiate their incomes. There was enhanced community awareness which in turn contributed to an increase in seeking for treatment across all categories of community based service providers. The narrative (Narratives 17–23) suggest improvement in health systems in which all key players in the service provision were working together, amongst themselves and with the communities. Overall, the referral system was perceived to have been enhanced.

## Limitations

1. The major limitation of this study is that it is couched in a model—the TEAM that was developed in a given social-cultural context. The results may therefore not be applicable in a different context.
2. We were focused on how the service providers and users perceived TEAM. We did not therefore compare and contrast the different service users and service providers on their perception of TEAM. We recommend that this should form part of the aims in a future study.

## Conclusion

Service users and service providers developed their own perceptions on TEAM and expressed them in the form of narratives. They were positive on TEAM. The perceptions and narratives corroborate existing Kenyan quantitative data on TEAM. Further, the narratives from both informal and formal service providers lend support to the WHO recommendation to involve all of them if the treatment gap is to be reduced. We have achieved our aims: (1) we have documented the perception of patients, service provides on their perception on TEAM and how team impacted the health system; (2) we have demonstrated corroborations between the perceptions and related Kenyan quantitative data on TEAM.

## Data Availability

The data for this paper will be made available upon written request to the corresponding author detailing the specific parts of the data to be shared and the intended purpose. The data legally belongs to Africa Mental Health Research and Training Foundation (AMHRTF). If for publication, the authorship will be agreed upfront using the AMHRTF data sharing policy. All ethical considerations on human subject data will apply.

## Abbreviations

mhGAP-IG: mental health treatment gap intervention guidelines
WHO: World Health Organization
LMICs: low and middle income countries
THPs: traditional health practitioners
CHWs: community health workers
FH: faith healers
TEAM: Multisectoral Stakeholder TEAM Approach to Scale-Up Community Mental Health in Kenya—Building on Locally Generated Evidence and Lessons Learned
FGDs: focus group discussions
IHPs: informal health providers
KIDs: key informant discussions
KIIs: key informant interviews
